# 

**Published:** 1881-12-20

**Authors:** 


☞ OUR JOURNAL for 1882 will in no respect fall behind its present high standard as a practical and progressive Journal, but will improve in all departments, furnishing a better Journal for the ensuing year than ever before presented.

Parties receiving the present number as a sample, are requested to subscribe. Try it for the next year, or for six months at least, and if you are not pleased with it, then drop it and take our hat.

### *RECEIPIED.*

1881.—Drs. J S Knott, S L Lockwood, T L Appleby, M N Odum, R M Canuth; J W McCaleb. E H Wright, H B Johnson, W T Gautier, S A Vinson, A C Crymes, W A Culbertson, W T Foute, J H McMullan, J B Foster, J F Earnest, N O Harris, E D Yates, J B Rutland, C S Priestly, J J Gage, F M Thomasson, J T Stephens.

1882.—Drs. C H Smith, W Barton, A Gullett. T P Oliver, L H Reed, J D Bowers, J C Beauchampe.

